# Rapid controlled release by photo-irradiation using morphological changes in micelles formed by amphiphilic lophine dimers

**DOI:** 10.1038/s41598-021-90097-7

**Published:** 2021-05-24

**Authors:** Masaaki Akamatsu, Kazuki Kobayashi, Hiroki Iwase, Yoshifumi Sakaguchi, Risa Tanaka, Kenichi Sakai, Hideki Sakai

**Affiliations:** 1 Department of Pure and Applied Chemistry, Faculty of Science and Technology, Tokyo University of Science, 2641 Yamazaki, Noda, Chiba 278-8510 Japan; 2 Neutron Science and Technology Center, Comprehensive Research Organization for Science and Society (CROSS), 162-1 Shirakata, Tokai, Ibaraki 319-1106 Japan; 3 Research Institute for Science and Technology, Tokyo University of Science, 2641 Yamazaki, Noda, Chiba 278-8510 Japan

**Keywords:** Self-assembly, Self-assembly

## Abstract

Photo-induced rapid control of molecular assemblies, such as micelles and vesicles, enables effective and on-demand release of drugs or active components, with applications such as drug delivery systems (DDS) and cosmetics. Thus far, no attempts to optimize the responsiveness of photoresponsive molecular assemblies have been published. We previously reported photoresponsive surfactants bearing a lophine dimer moiety that exhibit fast photochromism in confined spaces, such as inside a molecular assembly. However, rapid control of the micelle structures and solubilization capacity have not yet been demonstrated. In the present work, photo-induced morphological changes in micelles were monitored using in-situ small-angle neutron scattering (SANS) and UV/Vis absorption spectroscopy. An amphiphilic lophine dimer (3TEG-LPD) formed elliptical micelles. These were rapidly elongated by ultraviolet light irradiation, which could be reversed by dark treatment, both within 60 s. For a solution of 3TEG-LPD micelles solubilizing calcein as a model drug molecule, fluorescence and SANS measurements indicated rapid release of the incorporated calcein into the bulk solvent under UV irradiation. Building on these results, we investigated rapid controlled release via hierarchical chemical processes: photoisomerization, morphological changes in the micelles, and drug release. This rapid controlled release system allows for effective and on-demand DDS.

## Introduction

Amphiphilic compounds or surfactants form a variety of molecular assemblies, such as micelles, vesicles in water. The aqueous solutions of molecular assemblies can solubilize water-insoluble or poorly water-soluble compounds by incorporation into the molecular assemblies. This solubilization technique is used to dissolve active substances in aqueous media and is an indispensable step for the production of commercial products in the field of beverages, foods, cosmetics, medicines, and also household products.

**Figure 1:**
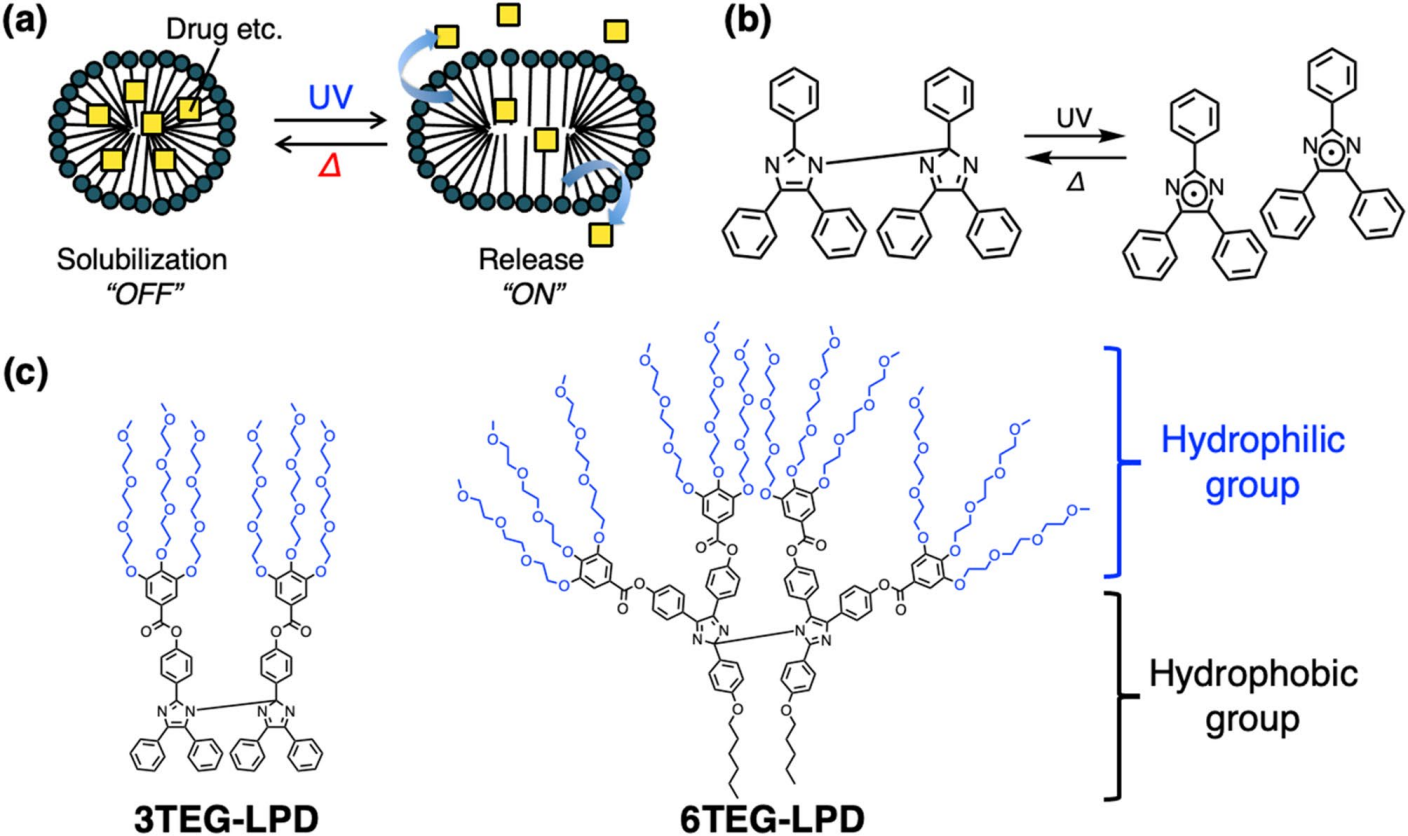
(**a**) Schematic image of controlled release with a photoresponsive molecular assembly. (**b**) Photochromism of the lophine dimer. (**c**) Chemical structures of the amphiphilic lophine dimers.

Controlled assembly of the amphiphilic compounds causing deformation and/or morphological changes can be applied for the regulated release of drugs and other active compounds (Fig. 1a). To trigger morphological changes in the molecular assemblies, external stimuli are applied, such as light, redox reaction, magnetic field, or temperature or pH change^1,2^. Among these, light is a promising external stimulus because it is clean and easily applied with high spatial resolution and specific wavelength. Therefore, many photoresponsive amphiphilic compounds have been developed, and the formation of molecular assemblies, such as micelles and vesicles, by photo-irradiation has already been realized^1,3,4^.

For decades, morphological changes in molecular assemblies by photo irradiation and their applications have been accomplished. For example, our group demonstrated controlled release of volatile oils as model perfumes with an azobenzene-type cationic surfactant that exhibits photoswitchable formation of micelles upon UV irradiation^5,6^. However, to induce significant changes, these conventional systems require minutes to hours of photo-irradiation. Considering practical applications, the response time needs to be fast, requiring a change in solution properties at an arbitrary moment (ON state) which ceases when the stimulus stops (OFF state) (Fig. 1a). To the best of our knowledge, there has been no attempt to speed up the controlled formation of photoresponsive molecular assemblies.

To achieve rapid control of amphiphile self-assembly, we focused on the lophine dimer (LPD) as a photochromic moiety. LPD molecules quickly dissociate into two lophyl radicals and are recovered by a thermal recombination reaction (Fig. 1b)^7^. Although this recombination is extremely slow, it has been reported that the reaction rate is significantly enhanced by inhibiting free diffusion of the radical species. This has been achieved by connecting two lophine moieties through a linker^8–10^, confining lophine dimers in micelles formed by surfactants^11–13^, or confining them to microscopic domains formed by ionic liquids^14,15^. We previously achieved rapid photoisomerization of the lophine dimer in micelles formed by amphiphilic lophine dimers themselves (3TEG-LPD, shown in Fig. 1c)^12^ and demonstrated rapid and reversible variations in the surface tension of the aqueous solution^13^. However, so far rapid morphological control of the micelles and controlled release system has not yet been accomplished. In the present study, we examine rapid morphological changes in micelles formed by amphiphilic lophine dimers and demonstrate controlled release of a model drug solubilized in the micelles. However, conventional particle-sizing equipment using light scattering and laser diffraction are problematic for the detailed morphological analysis of molecular assemblies and their changes under light irradiation.

Small-angle X-ray or neutron scattering (SAXS or SANS) provides precise and direct information about the structures of molecular assemblies. Another interesting feature of small-angle scattering is its capacity for in-situ and time-resolved measurement, allowing the monitoring of the dynamics of phase changes triggered by external stimuli^16–18^. Combining a light source and a spectrometer into a SAXS or SANS measurement system enables the monitoring of the morphological changes in globular to wormlike micelles formed by photoresponsive surfactants^19,20^. This analytical system is suitable for in-situ analysis of micelles formed by amphiphilic lophine dimers.

In this work, using a simultaneous SANS and Ultraviolet/Visible (UV/Vis) absorption measuring system, we performed in-situ observations of photo-induced morphological changes in micelles formed by two different amphiphilic lophine dimers (3TEG-LPD and 6TEG-LPD) in the order of several tens of seconds. Furthermore, the ability to control the release of a model drug by photo-irradiation was examined. From these experiments, we investigated rapid controlled release owing to hierarchical chemical process, consisting of photoisomerization, morphological changes in the micelles, and drug release.

## Results and discussion

**Figure 2:**
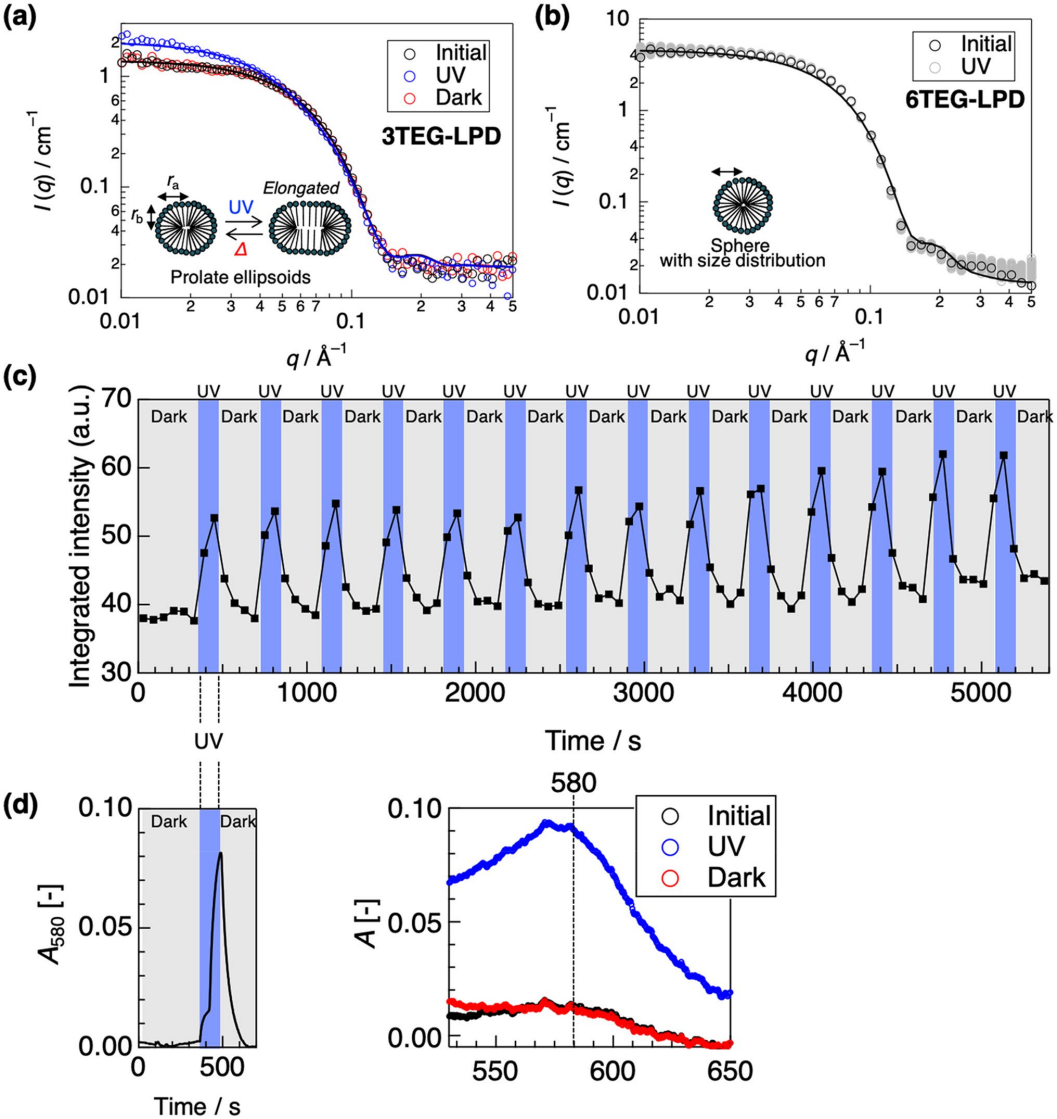
Results of simultaneous in-situ SANS and UV/Vis absorption. (**a**) SANS profiles of 10 mM 3TEG-LPD in D_2_O before and after 2 min UV irradiation and 4 min standing in the dark with curve fitting. (**b**) SANS profiles of 10 mM 6TEG-LPD in D_2_O before and after UV irradiation for 80 min with curve fitting. The gray plots are experimental SANS profiles during UV irradiation (**c**) The integrated scattering intensity of the SANS profiles of 3TEG-LPD in the *q*-region of 0.01–0.05 Å^−1^ during cycles of irradiation and standing in the dark. (**d**) UV/Vis absorption spectra before and after UV irradiation and standing in the dark (right), and the temporal changes in absorption at 580 nm during the cycle using an in-situ UV/Vis absorption spectrometer (left).

To reveal morphological changes in the micelles and the photoisomerization upon UV irradiation, we performed simultaneous in-situ SANS and UV/Vis absorption measurements by installing a mercury lamp and an UV/Vis absorption spectrometer on the sample table of a SANS instrument (Figure S1). Figure 2a shows the SANS profiles of 10 mM 3TEG-LPD in D_2_O. To perform SANS measurements at a sufficiently larger concentration than the cmc of 3TEG-LPD (0.80 μM^13^), the concentration of 3TEG-LPD was set at 10 mM. Several theoretical models, such sas uniform or polydisperse spheres and cylinders, were considered. The experimental data could be well fitted using a uniform prolate ellipsoid model^21^ with a long radius (*r*_a_) of ~ 47 Å and a short radius (*r*_b_) of ~ 28 Å (Fig. 2a, Table 1). UV irradiation caused an increase in the scattering intensity toward the lower *q* region, and this reached a constant state within 2 min. The post-irradiated SANS profile was also well fitted using a uniform prolate ellipsoid model. The short radius remained at ~ 28 Å and a long radius increased to ~ 70 Å, indicating that UV irradiation induced elongation of the longer axis of the micelles. This is because the hydrophobic portion of the resulting lophyl radical has a relatively larger volume in comparison with that of the original dimer form, whose LPD unit is tightly linked with a covalent bond, and photo-irradiation caused formation of micelles having a lower curvature^22^.

**Table 1:** SANS profiles with fitting results, after UV irradiation and standing in the dark, of 10 mM 3TEG-LPD (a) and 6TEG-LPD (b) solutions.

	(a) 3TEG-LPD			(b) 6TEG-LPD	
	Initial	UV	Dark		Initial
*ϕ*	0.00303	0.00300	0.00302	*ϕ*	0.0144
*r*_a_/Å	46.5	69.4	46.9	*r*/Å	27.6
*r*_b_/Å	27.6	27.6	27.6	*p*	0.15
*ρ*_m_/10^−6^ Å^–2^	0.772	0.763	0.785	*ρ*_m_/10^−6^ Å^−2^	1.03
*χ*^2^	4.68	3.89	6.01	*χ*^2^	3.29

Prolate ellipsoids for 3TEG-LPD and spheres with a Schultz size distribution for 6TEG-LPD were modeled. *ϕ* is the volume fraction, and *r*_a_ or *r*_b_ is the long or short radius of ellipsoids, respectively. *r* and *p* are the mean radius and polydispersity of the radius, respectively. *ρ*_m_ is the scattering length density of the micelle. Here, the scattering length density of the micelle or solvent (*ρ*_s_) was fixed at 6.30 × 10^−6^ Å^−2^ for D_2_O. *χ*^2^ is the chi-square value for fitting.

When the UV irradiation ceased, the SANS profile readily recovered to its initial state (Fig. 2a). Figure 2c shows the integrated scattering intensity in the *q*-region of 0.01–0.05 Å^−1^, for the scattering profiles at 60 s intervals during cycles of 2 min UV irradiation followed by 4 min standing in the dark (Figure S2). This observation revealed that the elongation of the elliptical micelles completed in 60 s of irradiation and reversed in 60 s of standing in the dark. These reversible changes in the SANS profiles were repeatable over 10 cycles, after which there was incomplete recovery due to partial decomposition. During the SANS measurements, UV/Vis absorption spectra were simultaneously collected. As shown in Fig. 2d, a characteristic absorption band of the lophyl radical at 580 nm was observed during UV irradiation. The temporal change in the absorption at 580 nm during the irradiation cycle revealed that morphological changes in the micelles and production of the lophyl radicals occurred without time lag within the analytical time resolution (Fig. 2c,d). These results indicate that the reorganization of the surfactant molecules in the micelles following photoisomerization is complete within the time scale of the SANS measurements.

Interestingly, the photoisomerization and morphological changes in the molecular assembly of the amphiphilic lophine dimers readily proceed even in highly concentrated systems, unlike azobenzene derivatives, which are the most popular photochromic compounds. Azobenzene derivatives show a lower rate and yield of photoisomerization in a confined system^23^ or in the solid state^24^ because sufficient free volume is necessary for conformational changes in the molecular structure during the *trans*–*cis* isomerization. Therefore, the lophine dimer analogue is suitable for photoresponsive functional systems based on molecular assemblies or supramolecules.

To examine the effects of the position of hydrophilic groups and the balance of hydrophilicity and lipophilicity on the speed of the recombination reaction and morphological changes in the micelle, 6TEG-LPD was synthesized according to Scheme S1. According to the static surface tension measurements of aqueous solutions of 6TEG-LPD, the critical micellar concentration (cmc) was 0.35 μM (Figure S3), which is lower than that of 3TEG-LPD (0.80 μM^13^). We assume that the introduction of alkyl chains promoted adsorption of 6TEG-LPD at the air/water interface and formation of the micelles. For the micellar solution of 6TEG-LPD, the dimer-monomer photoisomerization proceeded readily according to the UV/Vis absorption measurements (Figure S4). The apparent rate of the latter recombination of the lophyl radicals (*k*′), with the assumption of a second-order rate reaction, was 1.5 s^−1^, which was approximately a 1500-fold enhancement compared to that in tetrahydrofuran (THF), where 6TEG-LPD does not form micelles and the lophyl radicals freely diffuse. From this observation, 6TEG-LPD showed better enhancement in the radical recombination than 3TEG-LPD with an approximate 800-fold increase^13^, although there is almost no difference in the absolute values of the apparent recombination rates (*k*′) in the micellar solutions.

SANS data of 5.0 mM 6TEG-LPD in D_2_O were well fitted with a model of polydisperse spheres with an average radius (*r*) of ~ 27 Å (Fig. 2b). We assume that 6TEG-LPD bearing more bulky hydrophilic groups is likely to form micelles with larger curvature. However, the SANS profile was unchanged by UV irradiation for over 80 min (Fig. 2b). This suggests that UV irradiation induces no morphological change in 6TEG-LPD micelles, despite the photoisomerization proceeding under UV irradiation. We interpret that flexible alkyl chains compensate for UV-induced structural changes in the surfactant, explaining the nonresponsive behavior of the micellar morphology.

**Figure 3:**
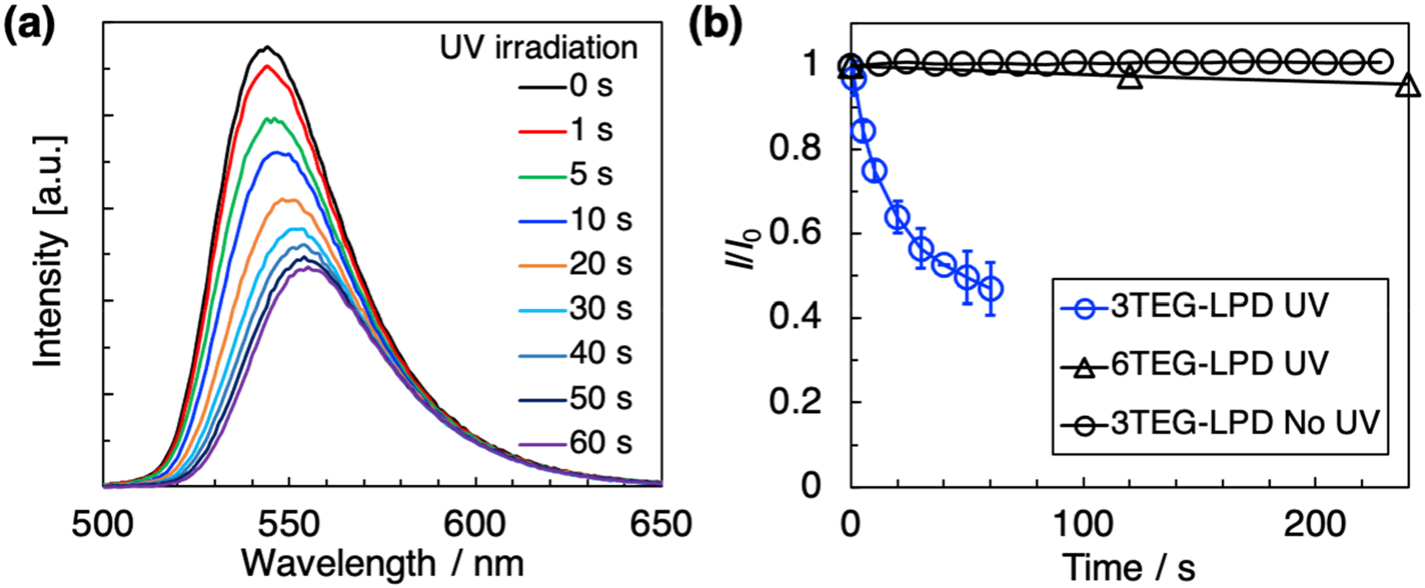
(**a**) Variations in fluorescence spectra of 1.0 mM calcein/5.0 mM 3TEG-LPD aqueous solution under UV irradiation. (**b**) The normalized transient changes in fluorescence intensity at the peaks for 1.0 mM calcein/5.0 mM 3TEG-LPD in the absence and presence of UV irradiation and 6TEG-LPD aqueous solutions in the presence of UV irradiation.

The abovementioned results show that UV irradiation induced rapid and reversible changes in the morphology of the 3TEG-LPD micelles. Using this promising photoresponsive surfactant, substances solubilized in the micelles can be readily released by UV irradiation. To demonstrate controlled release, we prepared an aqueous solution of 3TEG-LPD containing calcein as a fluorescent model drug. Figure 3a shows the fluorescence spectra of a 5.0 mM 3TEG-LPD solution with calcein at the solubilization limit (1.0 mM) as a function of UV irradiation time. Under UV irradiation, the band at 545 nm assigned to calcein in the micelles decreased, with the peak shifting to a band at 556 nm assigned to aggregated calcein molecules in water. This indicated calcein molecules incorporated in the micelles released under UV irradiation. Figure 3b shows the temporal changes of the relative intensity of the fluorescence peak. The intensity reached a constant state within 60 s of UV irradiation, which is slightly shorter than the change in the SANS profile. We assume that UV irradiation for 60 s induced effective morphological changes in the micelles, affecting the solubilization state of calcein. In contrast, the fluorescence spectra of the calcein/3TEG-LPD aqueous solution remained in the absence of UV irradiation (Figs. 3b and S5a), representing that UV light induced release of calcein from inside of the 3TEG-LPD. The fluorescence of calcein in the 6TEG-LPD micelles was unchanged upon UV irradiation (Figs. 3b and S5b), corresponding to the nonresponsive behavior of 6TEG-LPD in the SANS profile (Fig. 2b). Furthermore, the fluorescence spectra of aqueous calcein solution almost unchanged during UV irradiation (Figure S5c). These results indicate that the photo-bleaching of calcein was not responsible for the fluorescence changes.

**Figure 4:**
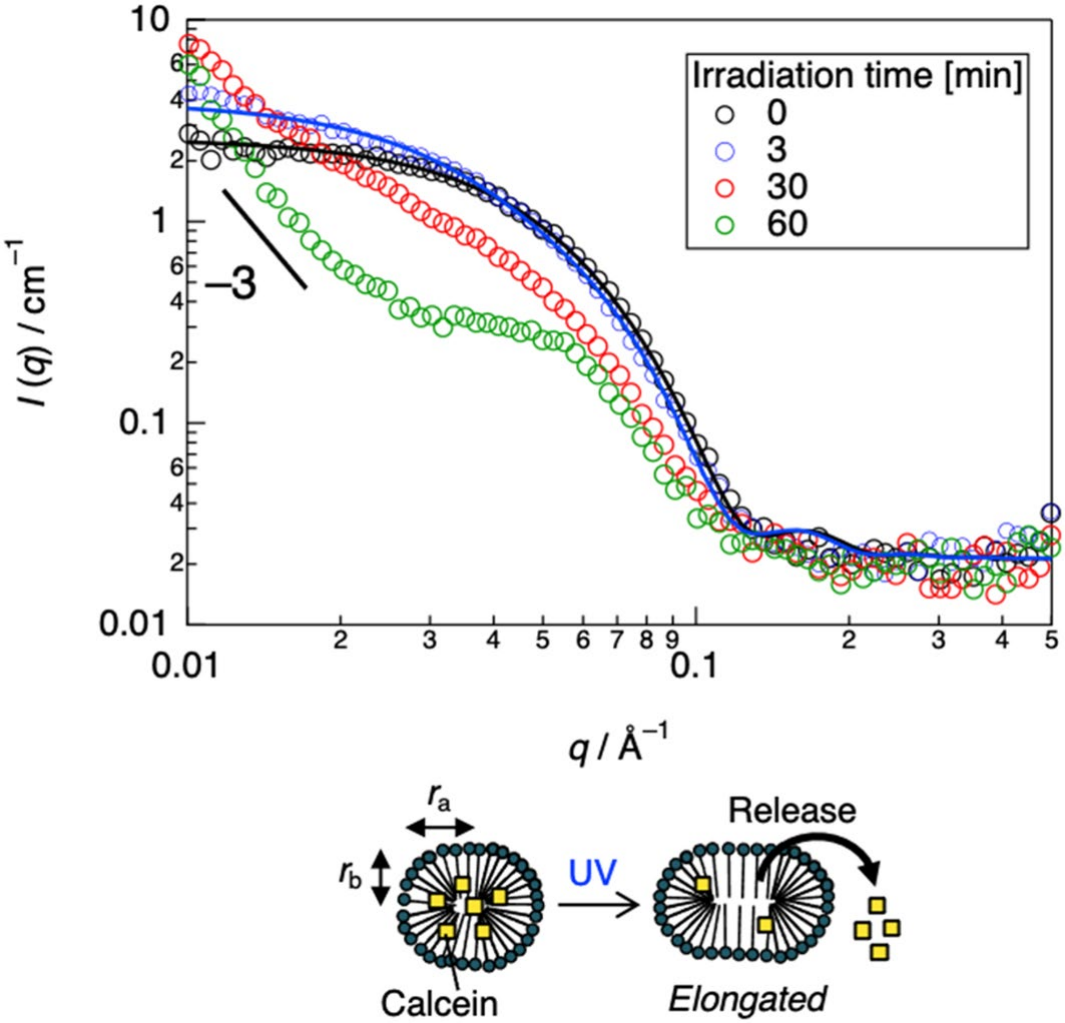
Variations in SANS profiles of 1.0 mM calcein/10 mM 3TEG-LPD D_2_O solution under UV irradiation with curve fitting.

To elucidate the changes in solubilization under UV irradiation, we also performed in-situ SANS on the solutions. As shown in Fig. 4, a SANS profile of 5.0 mM 3TEG-LPD in D_2_O with 1.0 mM calcein was well fitted using a model of a uniform prolate ellipsoid with a long radius (*r*_a_) of ~ 66 Å and a short radius (*r*_b_) of ~ 32 Å (Table 2), indicating that the 3TEG-LPD micelles swelled as a result of calcein incorporation owing to hydrophobic interaction and/or π–π interaction between aromatic groups of 3TEG-LPD and calcein. UV irradiation caused gradual changes in the SANS data. UV irradiation for 3 min produced a SANS profile similar to that of the empty 3TEG-LPD micelles under UV irradiation (Fig. 2a). Further irradiation induced a significant increase in the scattering profile in the lower *q*-range of < 0.02 Å^−1^ and the slope was *q*^–3^, indicating that coarse aggregates of surplus calcein formed, as visualized in Figure S7. In the SANS profile of 6TEG-LPD in D_2_O, calcein solubilization was unchanged by UV irradiation (Figure S6). These results reveal that the morphological change in the 3TEG-LPD micelles under UV irradiation caused rapid and effective release of calcein. This rapidly controlled release system allows for effective and on-demand delivery systems of active components, such as drugs and perfumes.

**Table 2:** SANS profiles with fitting results, after UV irradiation and standing in the dark, of 5.0 mM 3TEG-LPD (a) and 6TEG-LPD (b) solutions saturated with calcein.

	(a) 3TEG-LPD		(b) 6TEG-LPD	
	Initial	UV (2 min)		Initial
*ϕ*	0.00289	0.00276	*ϕ*	0.00972
*r*_a_/Å	66.0	98.3	*r*/Å	28.5
*r*_b_/Å	31.9	33.1	*p*	0.13
*ρ*_m_/10^–6^ Å^−2^	0.678	0.713	*ρ*_m_/10^–6^ Å^−2^	1.96
*χ*^2^	3.83	5.59	*χ*^2^	3.81

Prolate ellipsoids for 3TEG-LPD and spheres with a Schultz size distribution for 6TEG-LPD were modeled.

## Conclusions

To investigate photo-induced rapid controlled release systems, we developed photoresponsive surfactants bearing a lophine dimer moiety that proceeds with fast photochromism in confined spaces such as inside molecular assemblies. Rapid control of micelle structures and solubilization capacity have not been accomplished yet. An in-situ SANS system simultaneously incorporating a light source and a UV/Vis absorption spectrometer showed that the morphological changes in micelles formed by the amphiphilic lophine dimer (3TEG-LPD) and the photochromism between dimer and radical forms proceeded within several tens of seconds. On the other hand, another surfactant bearing alkyl chains (6TEG-LPD) showed no morphological change in the micelles under UV irradiation. We assume that the flexible alkyl chains compensate for the change in the hydrophobic section of 6TEG-LPD radicals.

We studied the controlled release of calcein as a model drug using micellar solutions of amphiphilic lophine dimers. The fluorescence spectra revealed that UV irradiation caused the release of calcein from inside the micelles within 60 s, which is faster than the morphological change in the empty micelles. This result indicates that the change in the solubilization of calcein under UV light is faster than the completion of morphological changes in the micelles. In-situ SANS revealed similar morphological changes in the calcein-incorporating micelles and in the empty micelles.

In the previous studies, we demonstrated rapid photoisomerization with the lophine dimer in micelles and control of surface tension of the aqueous solution. In the present study, rapid morphological controls of the micelles and controlled release system were accomplished by using the amphiphilic lophine dimer. From these results, rapid controlled release via hierarchical chemical processes: photoisomerization, morphological changes in the micelles, and drug release was investigated. This rapidly controlled release system allows for effective and on-demand delivery systems of drugs, perfumes, or other active components. Currently, to avoid damages of these guest molecules and organisms by the UV irradiation, we are developing molecular assemblies responding red or near infrared light. Furthermore, we are working on application of fast morphological control of molecular assemblies to wormlike micelles that have characteristic viscoelasticity in solution, owing to a polymer-like network, which can be applied in control of texture, heat exchange, etc. Therefore, this study could contribute to medicine, cosmetics, food, and device development.

## Methods

### Materials

Solvents and reagents were purchased from Tokyo Chemical Industry Co. Ltd. (Tokyo, Japan) and FUJIFILM Wako Pure Chemical Co. (Osaka, Japan) and used without further purification. All reaction mixtures and fractions eluted by column chromatography were monitored using thin layer chromatography (TLC) plates (Merck, Kieselgel 60 F254). The TLC plates were observed under UV light at 254 and 365 nm. Flash column chromatography over silica gel (Wakosil C-200, 64–201 μm) was used for separation.

### Measurements

^1^H- and ^13^C-NMR spectra were measured at 298 K in a DMSO-*d*_6_ solution of the samples, using a JEOL model JNM-AL300 (300 MHz) or JNM-ECP500 (500 MHz) spectrometer with Si(CH_3_)_4_ as an internal standard. Chemical shifts (*δ*) and coupling constants (*J*) are reported in parts per million (ppm) and Hertz (Hz), respectively. Electrospray ionization mass spectrometry (ESI–MS) spectra were recorded using a JMS-T100CS instrument (JASCO, Tokyo, Japan). High-resolution mass spectrometry (HRMS) spectra (ESI-negative) were recorded using a JMS-MS700 system (JEOL, Tokyo, Japan). Elemental analysis was performed using a PE 2400II (PerkinElmer, Massachusetts, US) system. UV/Vis absorption spectra were measured using an Agilent 222 UV/Vis spectrophotometer with a quartz cuvette (1.0 cm path length).

### Synthesis and the structural characterization

Synthesis of 3TEG-LPD was performed according to a previously reported procedure^13^. A synthetic route for 6TEG-LPD is shown in the supplementary information.

### Surface and interfacial tension measurements

Static surface tension was measured on a platinum plate at room temperature (25.0 °C) using a K100 auto surface tensiometer (Krüss, Hamburg, Germany). The surface tension was assumed to be equilibrated when the value became constant.

### Photoisomerization of the amphiphilic lophine dimers

UV irradiation was performed using a 200 W Hg-Xe Lamp (SUPERCUR UVF-203S, SAN-EI ELECTRONIC). The irradiation wavelength (260–390 nm) was achieved using a color filter (U340, HOYA). Each solution was irradiated with light (20.0 mW/cm^2^) in a 1.0 cm path quartz cuvette. The photochromic behavior of 6TEG-LPD was characterized by UV/Vis absorption spectroscopy (Agilent 222). The apparent rates for the latter recombination of the lophyl radicals (*k*′) were calculated from the slope of the reciprocal of absorbance at 620 nm originating from the lophyl radical vs. time plots.

### In-situ SANS

Small-angle neutron scattering (SANS) measurements were performed using TAIKAN on the BL15 beamline at the Material and Life Science Experimental Facility (MLF) in the Japan Proton Accelerator Research Complex (J-PARC)^25^. The neutron wavelength was set to 0.8–7.8 Å. The sample-to-detector distance was 5.65 m. The *q* range was from 0.01 to 1.0 Å. The *q* value is defined by *q* = 4πsin(*θ*/2)/*λ*, where *θ* and *λ* are the scattering angle and wavelength, respectively. The samples were analyzed in a quartz cuvette of 2.0 mm thickness. SANS measurements were performed at 25 °C. The intensity of the scattering profile was converted to absolute intensity by subtraction with a profile of D_2_O and standardization with a profile of glassy carbon. Data reduction and analysis were performed with Igor Pro (Wave-metrics Inc., version 8.03) using the NIST reduction and analysis macros (version 7.99)^26^.

The set-up of the in-situ SANS apparatus is shown in Figure S1^27^. REX-250 (Asahi Spectra Co., Ltd.) was used as a UV lamp (365 nm, 26.2 mW/cm^2^). The UV/Vis absorption system consisted of a C10082CAH spectroscope and an L10290 light source (Hamamatsu Photonics K.K.). The UV light was reflected with a mirror and directed into a sample cuvette. Neutrons could penetrate the mirror. The light for UV/Vis absorption spectroscopy was passed through the upper part of the sample cuvette. A photo detector was installed to observe the exact time at which the light was emitted. During light irradiation, temperature changes in the samples were negligible.

In general, the scattering function for an assembly of particles is given by

1 $$I\left(q\right)={N}_{p} \cdot {\left(\Delta \rho \right)}^{2} \cdot {{V}_{p}}^{2} \cdot P(q) \cdot S(q)$$

where *N*_p_, $\Delta \rho$, and *V*_p_ are the number of particles per unit volume in solution, ﻿the scattering contrast, and the volume of a single particle, respectively. *P*(*q*) and *S*(*q*) are the form factor and structure factor, respectively. We employed the scattering functions of uniform ellipsoid and polydispersed sphere models.

For a prolate ellipsoid with minor axis *R*_1_ and major axis *R*_2_, the form factor *P*(*q*) is given by

2 $$P\left(q\right)=9{\int }_{0}^{1}{\left(\frac{{J}_{1}\left(q{R}_{s}\right)}{q{R}_{s}}\right)}^{2}dx$$

where *J*_1_(*x*) is the first order Bessel function of *x*. *R*_s_ is given by

3 $${R}_{s}={R}_{1}{\left[1+{x}^{2}\left({u}^{2}-1\right)\right]}^{1/2}$$

where u is the axis ratio (*R*_2_/*R*_1_).

For a polydispersed sphere model, the form factor *P*(*q*) is given by

4 $$P\left(q\right)={\int }_{0}^{\infty }{\left[\frac{3\left[\mathrm{sin}qr-(qr)\mathrm{cos}qr\right]}{{(qr)}^{3}}\right]}^{2}D(r)dr$$

where *r* is the mean radius of the micelle. The volume distribution function *D*(*r*) is represented by a Schulz distribution using the mean particle radius (*r*_m_):

5 $$D(r)={\left(Z+1\right)}^{Z+1}{\left(\frac{r}{{r}_{m}}\right)}^{Z}\frac{exp\left[-\frac{(Z+1)r}{{r}_{m}}\right]}{{r}_{m}\Gamma (Z+1)}$$

where *Γ*(*Z*) is the gamma function and *Z* is given by

6 $$Z = {\left( {{{{r_m}}/p}} \right)^2} - 1$$

Here, *p* represents the polydispersity index.

### Controlled solubilization upon UV irradiation with a fluorometer

1.0 mM of calcein was added to 5.0 mM aqueous 3TEG-LPD or 6TEG-LPD solution and stirred in the dark for more than two days to reach equilibrium. The 3TEG-LPD solution solubilizing calcein was added to a quartz cuvette of 1.0 cm thickness. Upon UV irradiation (260–390 nm, 20.0 mW/cm^2^), changes in fluorescence originating from calcein were monitored using an RF-5300PC (Shimadzu Corporation).

## Supplementary Information

Supplementary Information.
