# Supplementary material for: Rapid controlled release by photo-irradiation using morphological changes in micelles formed by amphiphilic lophine dimers
Source: Sci Rep. 2021 May 24;11:10754. doi: 10.1038/s41598-021-90097-7 (PMC8144387; doi:10.1038/s41598-021-90097-7)
Supplement: Supplementary file 1 — Supplementary Information. [file 41598_2021_90097_MOESM1_ESM.pdf]

**Rapid Controlled Release by Photo-Irradiation Using Morphological Changes in Micelles Formed by Amphiphilic Lophine Dimers**

Masaaki Akamatsu[1]*, Kazuki Kobayashi[1], Hiroki Iwase[2], Yoshifumi Sakaguchi[2], Risa Tanaka[1], Kenichi Sakai[1,3], Hideki Sakai[1,3]*

1) Department of Pure and Applied Chemistry, Faculty of Science and Technology, Tokyo University of Science, 2641 Yamazaki, Noda, Chiba 278-8510, Japan
2) Neutron Science and Technology Center, Comprehensive Research Organization for Science and Society (CROSS), 162-1 Shirakata, Tokai, Ibaraki 319-1106, Japan
3) Research Institute for Science and Technology, Tokyo University of Science, 2641 Yamazaki, Noda, Chiba 278-8510, Japan

Corresponding Authors' e-mail addresses: makamatsu@rs.tus.ac.jp, hisakai@rs.tus.ac.jp

## Supplementary Information

## 1. Synthesis and the structural characterization

A synthesis of 3TEG-LPD was performed according to the reported procedure[1]. Synthetic routes for 6TEG-LPD is shown in Scheme S1. **1**, **2**, **4** as intermediates were prepared according to the literature[1–3].

**Scheme S1**. Synthesis of 6TEG-LPD.

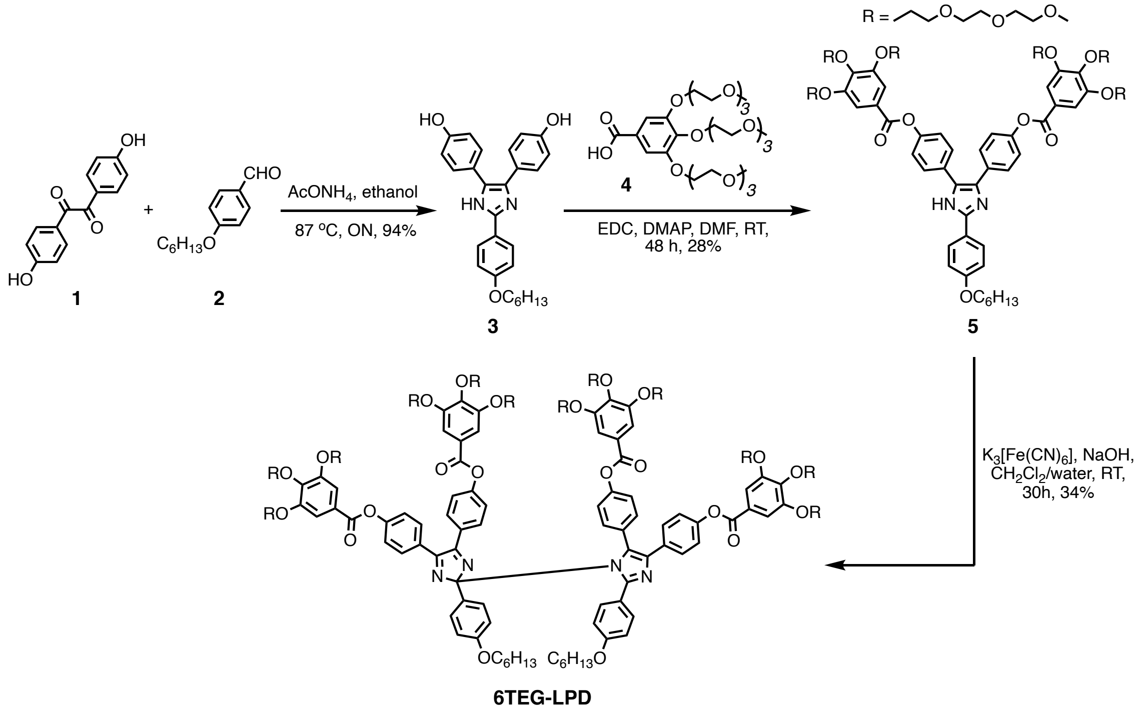


### Synthesis of 3

A 50 mL flask was charged with 1 (1.39 g, 5.70 mmol), 2 (1.17 g, 5.70 mmol), and ammonium acetate (3.18 g, 41.0 mmol) in ethanol (30 mL) and the mixture was heated at 87 °C for 12 h. Solvent was reduced under reduced pressure. The mixture was added into 300 mL of water. The resulting mixture was filtered and dried at reduced pressure. The pure product 3 was obtained as a white solid (94%). $^{1}$H-NMR (500 MHz, DMSO-$d_6$, 25 ºC): $\delta$ = 12.19 (s, 1H), 9.25 (s, 2H), 7.95 (d, $J$ = 9.1 Hz, 2H), 7.30 (m, 4H), 6.99 (d, $J$ = 9.1 Hz, 2H), 6.74 (m, 4H), 4.00 (t, $J$ = 6.5 Hz, 2H), 1.77–1.68 (m, 2H), 1.43 (t, $J$ = 7.8 Hz, 2H), 1.32 (m, 4H), 0.89 (t, $J$ = 6.9 Hz, 3H) ppm. $^{13}$C-NMR (125 MHz, DMSO-$d_6$, 25 °C): $\delta$ = 191.3, 172.3, 158.6, 144.6, 136.2, 132.3, 131.9, 129.7, 128.2, 126.5, 123.4, 115.4, 114.9, 114.8, 114.5, 67.5, 31.1, 28.7, 25.3, 22.1, 21.3, 14.0 ppm. MS (ESI): $m/z$ = 429.2 $[M+Na]^+$.

### Synthesis of 5

In a 300 mL round-bottomed flask, **3** (1.99 g, 4.652 mmol), **4** (6.10 g, 10.0 mmol),

*N*,*N*-dimethyl-4-aminopyridine (0.122 g, 1.00 mmol) and anhydrous *N*,*N*-dimethylformamide (60 mL) was stirred at RT. Then, 1-(3-dimethylaminopropyl)-3-ethylcarbodiimide hydrochloride (EDC·HCl) was added and stirred at RT under argon atmosphere for 48 h. The mixture was washed 2 times with water. The organic layer was dried by sodium sulfate and the solvent was removed under reduced pressure. The crude product was subjected to silica gel column chromatography (ethyl acetate/methanol = 15/1, v/v). to yield pure product **5** as a yellow oil (28%). $^{1}$H-NMR (500 MHz, DMSO-$d_6$): $\delta$ = 12.61 (s, 1H), 8.01 (d, $J$ = 9.1 Hz, 2H), 7.62 (t, $J$ = 8.9 Hz, 4H), 7.43 (d, $J$ = 5.4 Hz, 4H), 7.37 (d, $J$ = 8.7 Hz, 2H), 7.22 (d, $J$ = 8.7 Hz, 2H), 7.05 (d, $J$ = 8.7 Hz, 2H), 4.18 (q, $J$ = 5.2 Hz, 12H), 4.03 (t, $J$ = 6.4 Hz, 2H), 3.78 (s, 8H), 3.70 (t, $J$ = 4.7 Hz, 4H), 3.63-3.49 (m, 36H), 3.41 (m, 12H), 3.23 (d, $J$ = 5.1 Hz, 18H), 1.73 (q, $J$ = 7.0 Hz, 2H), 1.44 (t, $J$ = 8.0 Hz, 2H), 1.33 (d, $J$ = 4.0 Hz, 4H), 0.89 (t, $J$ = 7.1 Hz, 3H) ppm. $^{13}$C-NMR (125 MHz, DMSO-$d_6$, 25 °C): $\delta$ = 191.3, 172.3, 158.6, 144.6, 136.2, 132.3, 131.9, 129.7, 128.2, 126.5, 123.4, 115.4, 114.9, 114.8, 114.5, 67.5, 31.1, 28.7, 25.3, 22.1, 21.3, 14.0 ppm. MS (ESI): $m/z$ = 1631.7 $[M+Na]^+$. HRMS (ESI) calcd. for $C_{83}H_{120}N_2NaO_{29}$: 1631.78744, found: 1631.78641.

**Synthesis of 6TEG-LPD**

In a 300 mL round-bottomed flask, a mixture of **5** (0.955 g, 0.593 mmol), potassium ferricyanide (9.77 g, 29.7 mmol) and dichloromethane (150 mL) was stirred at room temperature for 0.5 hour. 100 mL of aqueous potassium hydroxide solution (3.33 g, 59.3 mmol) was added slowly to the mixture and stirred for 0.5 h. The reaction mixture was washed with water for 3 times and the organic layer was dried by sodium sulfate. After evaporating the solvent, the crude product was subjected to silica gel column chromatography (dichloromethane/methanol = 20/1, v/v) to yield pure product **6TEG-LPD** as a green oil (34%). $^{1}$H-NMR (500 MHz, DMSO-$d_6$): $\delta$ = 8.39 (d, $J$ = 8.0 Hz, 2H), 7.99 (dd, $J$ = 30.1, 8.3 Hz, 2H), 7.70-7.60 (m, 2H), 7.48-7.29 (m, 14H), 7.25–7.04 (m, 8H), 6.95–6.86 (m, 2H), 6.74–6.59 (m, 2H), 4.18–4.11 (m, 28H), 3.73 (d, $J$ = 33.8 Hz, 24H), 3.60–3.55 (m, 24H), 3.54–3.47 (m, 48H), 3.43–3.37 (m, 24H), 3.24–3.20 (m, 36H), 1.80–1.62 (m, 4H), 1.45–1.26 (m, 12H), 0.86 (d, $J$ = 37.2 Hz, 6H). $^{13}$C-NMR (125 MHz, DMSO-$d_6$, 25 °C): $\delta$ = 164.20, 164.16, 158.98, 152.18, 150.11, 149.27, 145.88, 142.67, 142.59, 136.17, 133.05, 129.59, 128.87, 128.01, 126.97, 126.74, 123.59, 123.46, 122.87, 122.33, 121.76, 114.59, 108.87, 108.81, 72.05, 71.3, 69.99, 69.95, 69.89, 69.82, 69.64, 68.97, 68.67, 67.55, 58.04, 31.05, 28.71, 25.24, 22.12, 13.93 ppm. ESI-MS: $m/z$ = 3261.5 $[M+Na]^+$. HRMS (ESI) calcd. for $C_{166}H_{238}N_4Na_2O_{58}$: 3261.55923, found: 3261.55922. IR (ATR): 1298 $cm^{-1}$ (C-N stretching vibration). Calculated for $C_{166}H_{238}N_4O_{58}$: %C, 61.96; %H, 7.46; %N, 1.74. Found: %C, 61.76; %H, 7.16; %N, 1.69.

## 2. Supplementary figures

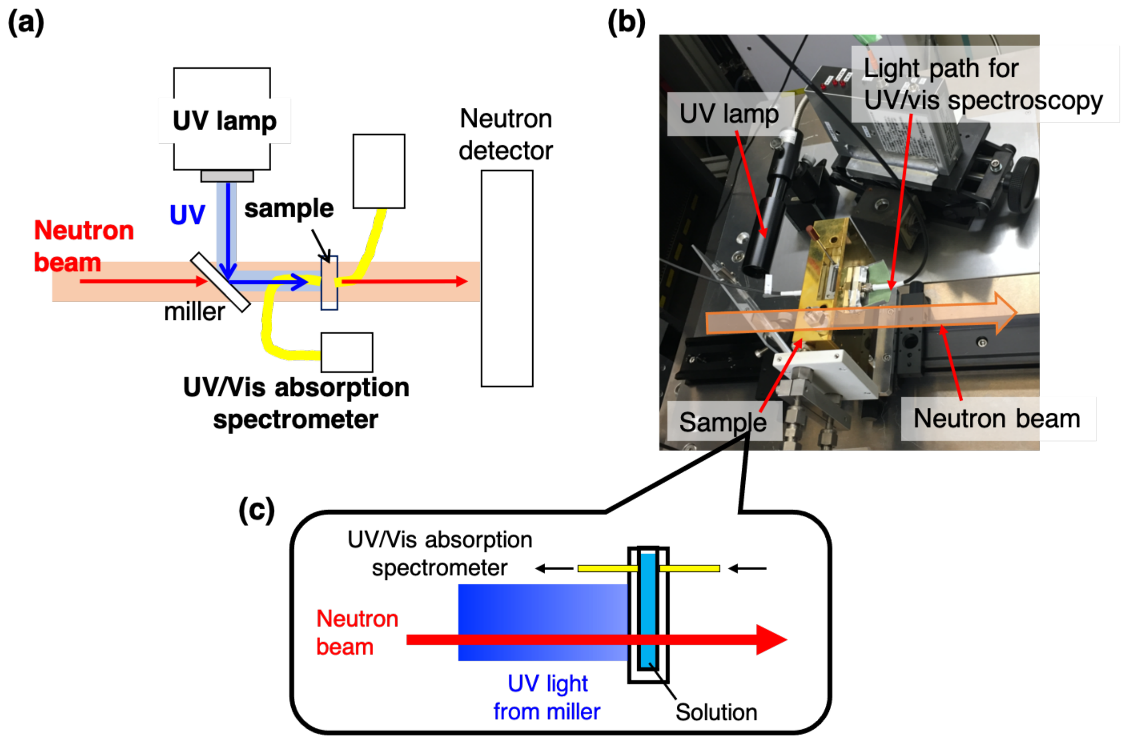


**Figure S1**. Schematic image and picture of *in-situ* SANS system (a and b). The detailed schematic image around the sample cuvette (c).

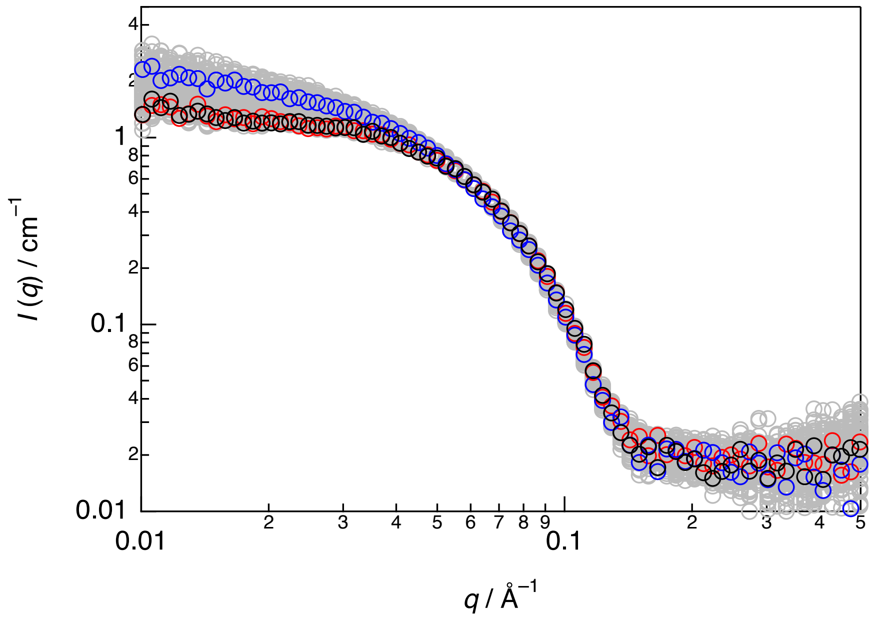


**Figure S2**. The transient variations of SANS profiles of 5.0 mM 3TEG-LPD in $D_2O$ under cycles of 2 min UV light irradiation and 4 min dark.

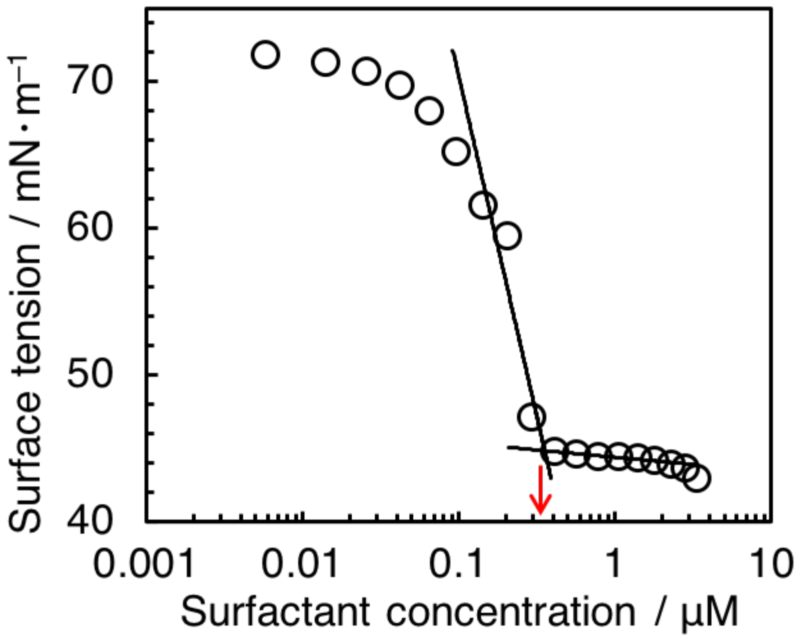


**Figure S3**. Static surface tension of aqueous 6TEG-LPD solutions measured at various surfactant concentrations at 25 °C.

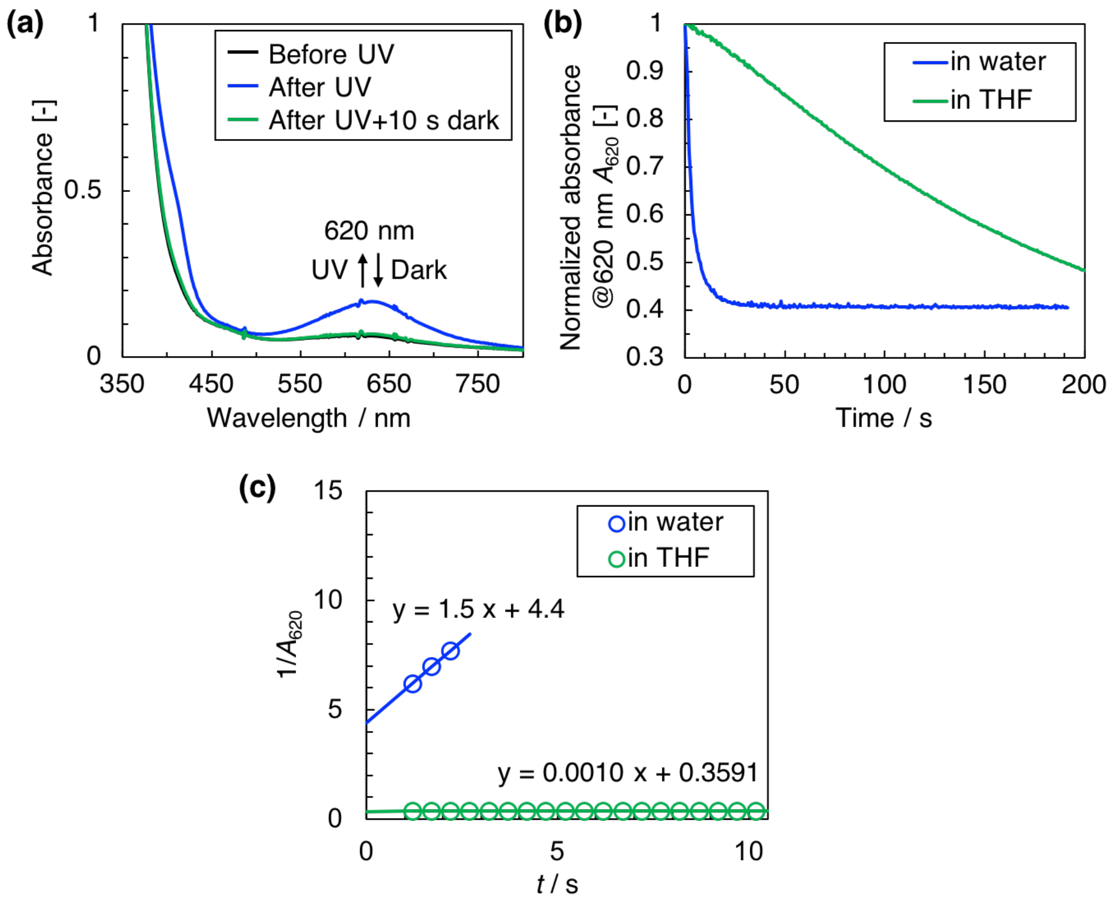


**Figure S4**. (a) Variations in the UV-vis absorption spectra of the 0.5 mM 6TEG-LPD aqueous solution. (b) The transient changes in absorption at 620 nm due to the lophyl radical production by UV irradiation in water or THF. (c) Plots of 1/*A* vs. time of the aqueous solutions of 0.5 mM 6TEG-LPD in water or THF. $A_{620}$ is the absorbance at 620 nm.

**Table S1**. Recombination rates of 3TEG-LPD or 6TEG-LPD in water or THF.

| | 3TEG-LPD[1] | 6TEG-LPD |
|---|---|---|
| Water | 1.4 | 1.5 |
| THF | 0.0018 | 0.0010 |

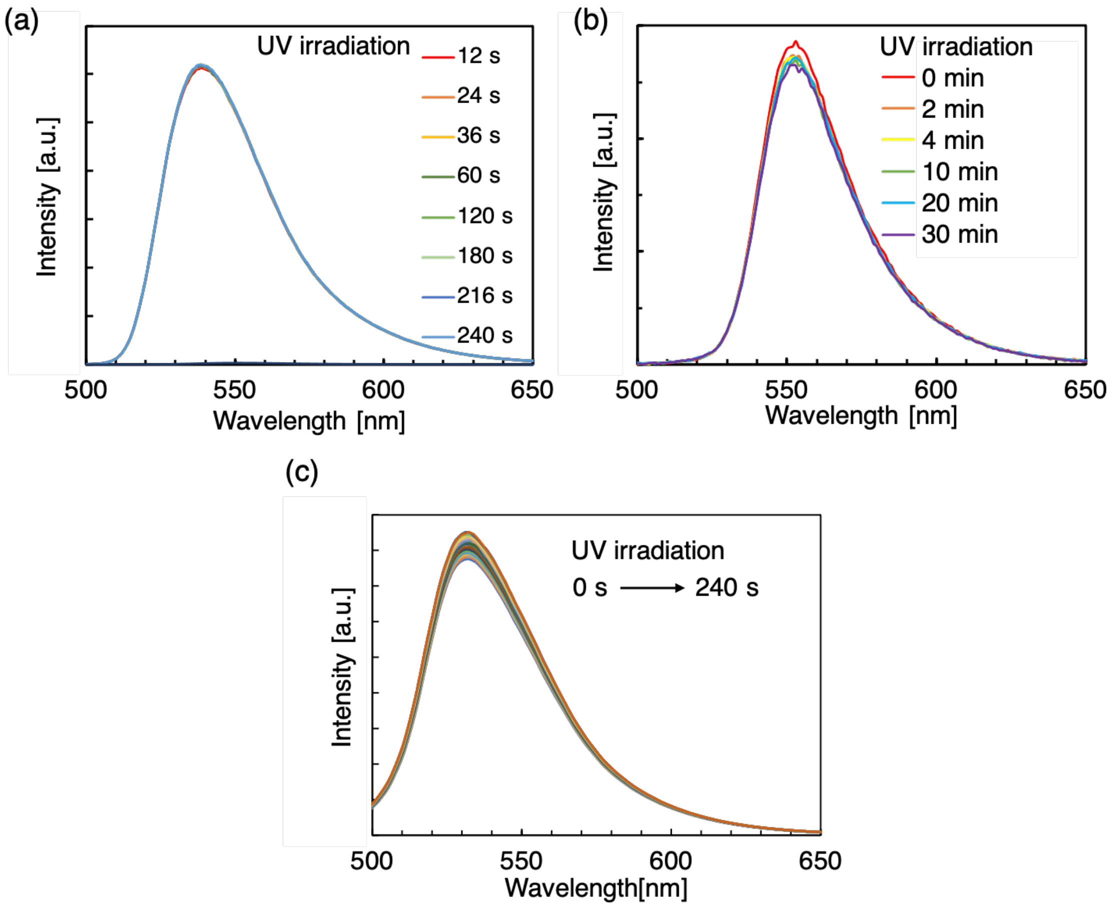


**Figure S5**. Variations in fluorescence spectra of 1.0 mM calcein/5.0 mM 3TEG-LPD aqueous solution in the absence of UV light irradiation (a) and 1.0 mM calcein/5.0 mM 6TEG-LPD aqueous solution in the presence of UV light irradiation (b), and 0.8 mM calcein aqueous solution in the presence of UV light irradiation (c).

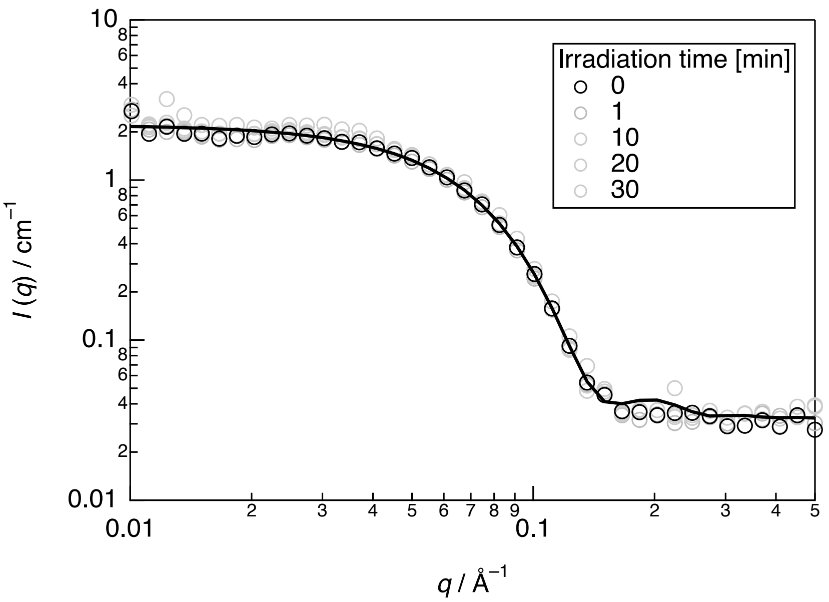


**Figure S6**. SANS profiles of 5.0 mM 6TEG-LPD in $D_2O$ before and after UV light irradiation.

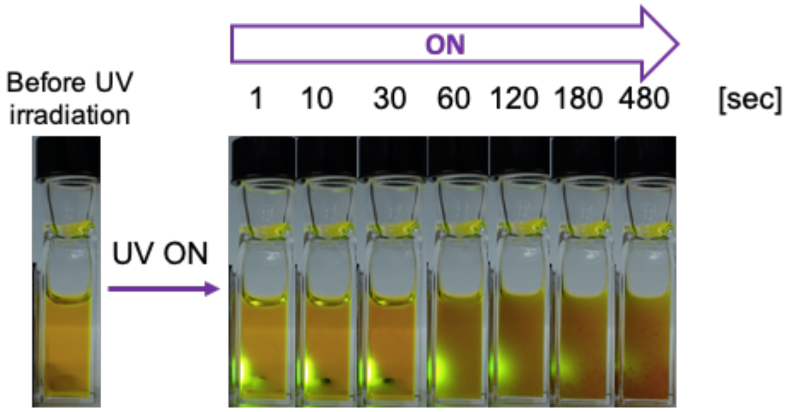


**Figure S7**. Appearances of calcein/5.0 mM 3TEG-LPD in water before and after UV light irradiation.

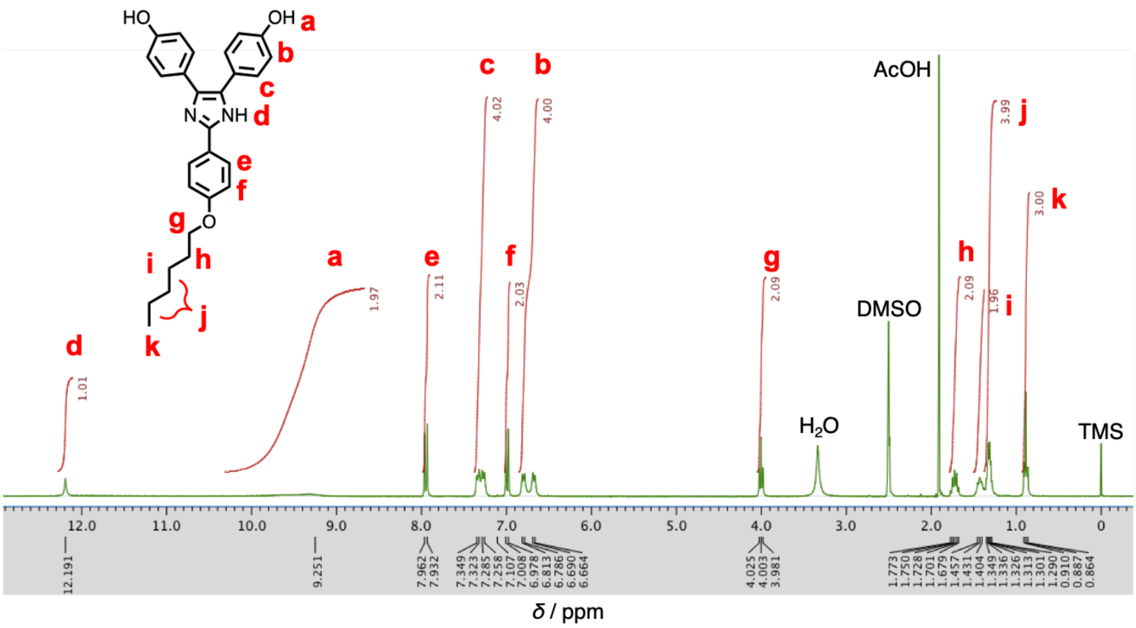


**Figure S8**. $^{1}$H-NMR spectrum of **3** in DMSO-$d_6$.

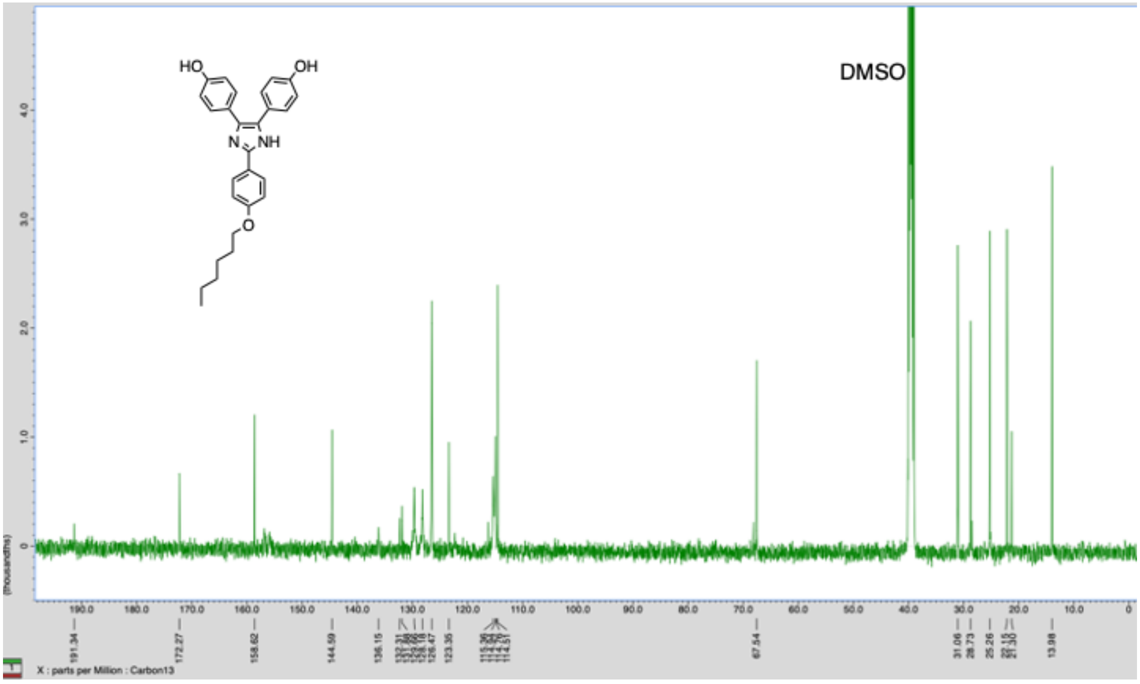


**Figure S9**. $^{13}$C-NMR spectrum of **3** in DMSO-$d_6$.

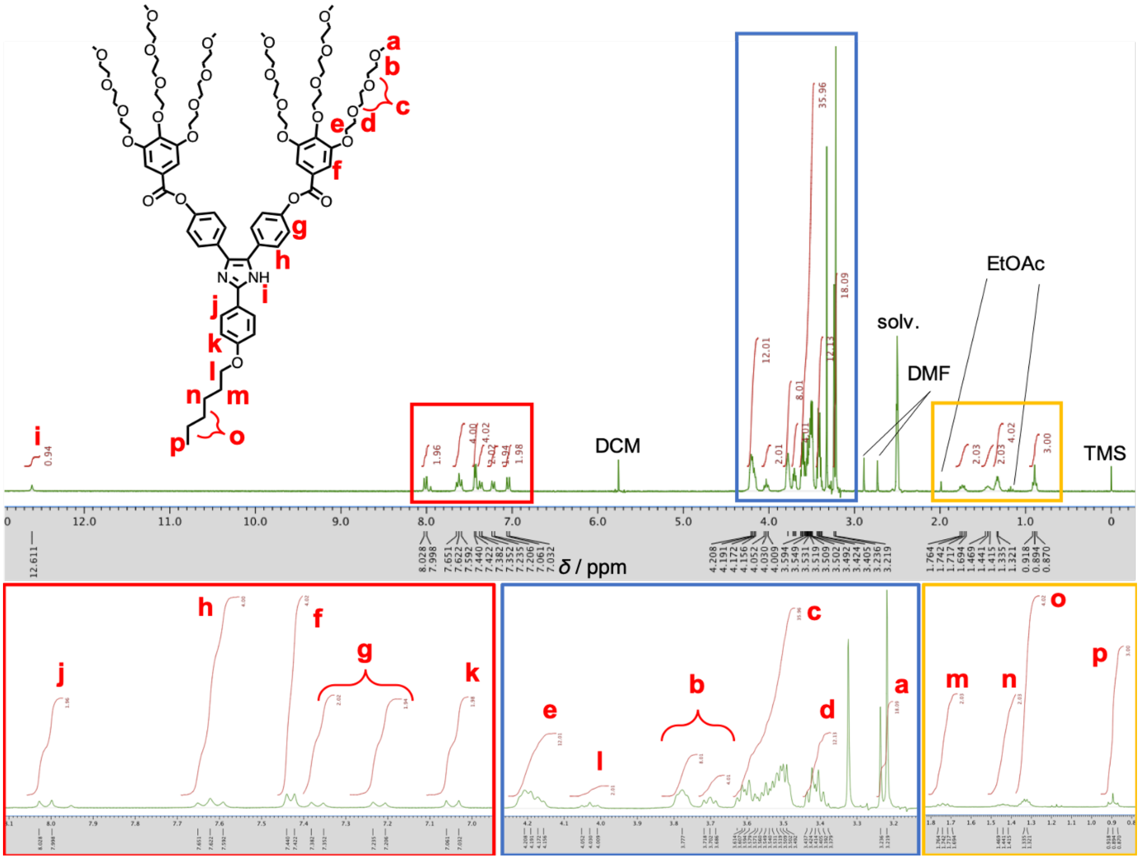


**Figure S10**. $^{1}$H-NMR spectrum of **5** in DMSO-$d_6$.

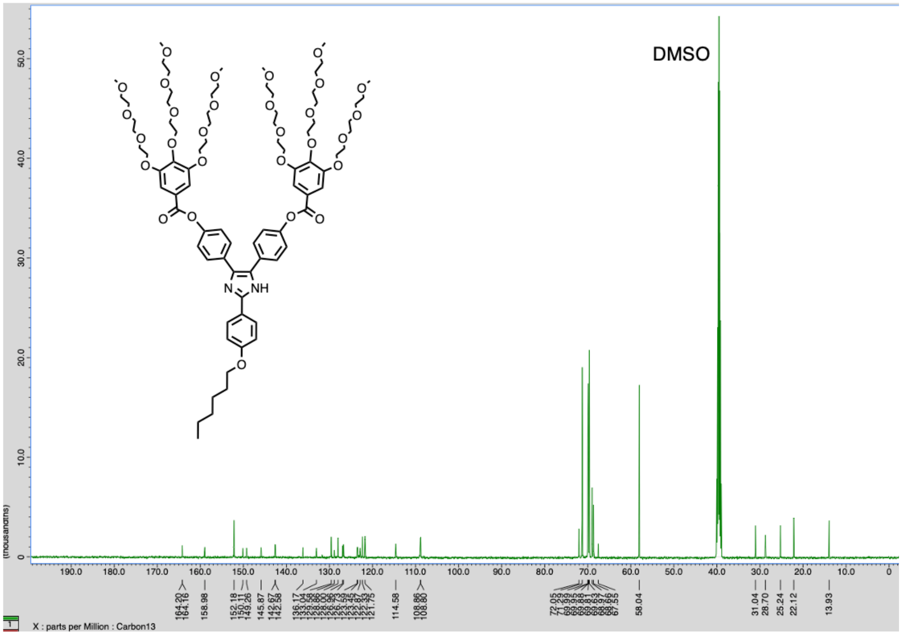


**Figure S11**. $^{13}$C-NMR spectrum of **5** in DMSO-$d_6$.

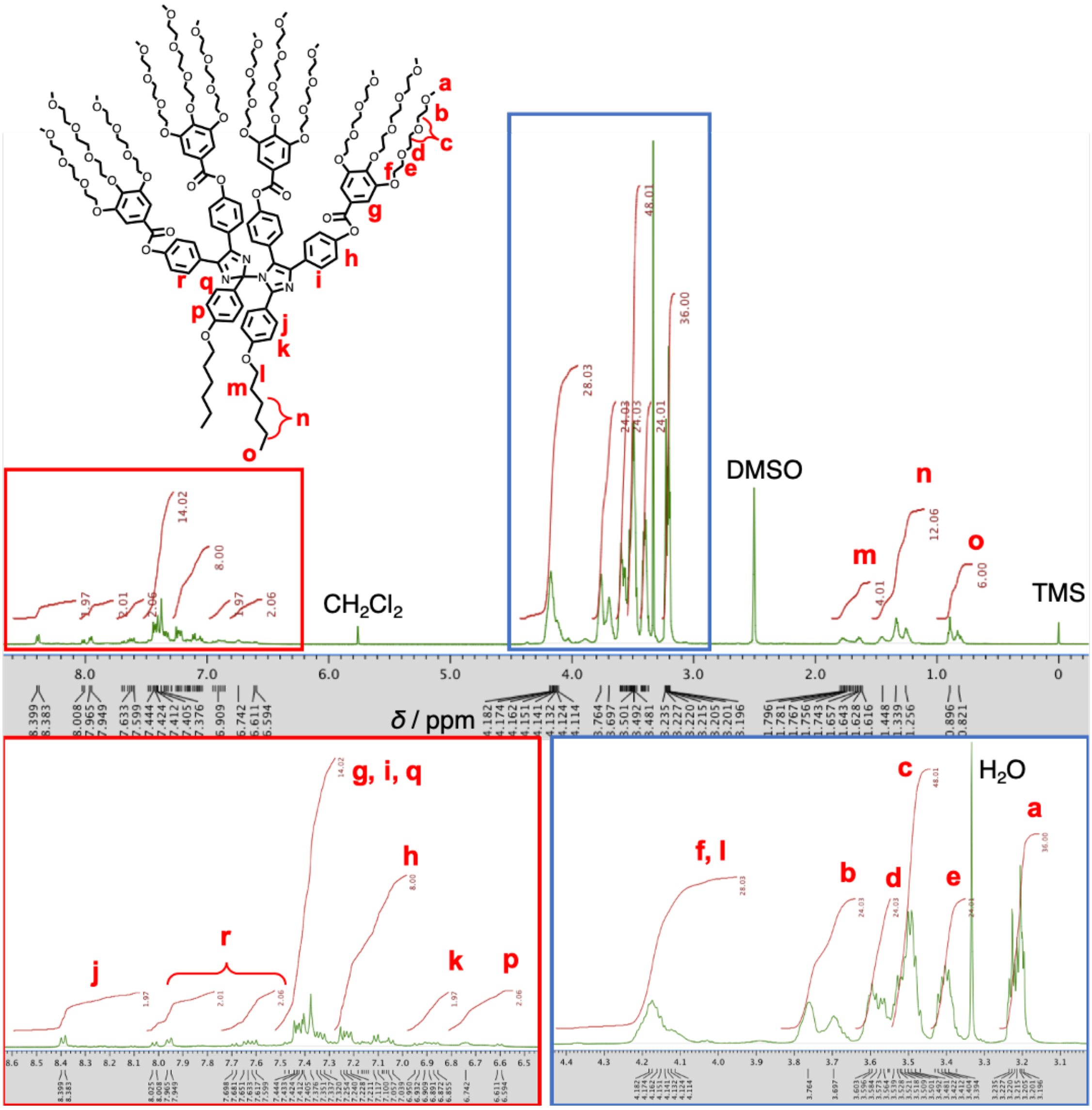


**Figure S12**. $^1$H-NMR spectrum of **6TEG-LPD** in DMSO-$d_6$.

**References**

1. Akamatsu, M., Kobayashi, K., Sakai, K. & Sakai, H. Accelerated recombination of lophyl radicals and control of the surface tension with amphiphilic lophine dimers. *Chem. Commun.* **55**, 9769–9772 (2019).
2. Bui, T. T., Garreau-De Bonneval, B. & Moineau-Chane Ching, K. I. Synthesis and preliminary physical properties of new neutral tetraalkoxy-substituted nickel bis(1,2-dithiolene) complexes. *New J. Chem.* **34**, 337–347 (2010).
3. Akamatsu, M. *et al.* Accelerated Recombination of Lophyl Radicals Solubilized in Micelles. *Chem. Lett.* **47**, 113–115 (2018).
